# TDNAscan: A Software to Identify Complete and Truncated T-DNA Insertions

**DOI:** 10.3389/fgene.2019.00685

**Published:** 2019-07-25

**Authors:** Liang Sun, Yinbing Ge, J. Alan Sparks, Zachary T. Robinson, Xiaofei Cheng, Jiangqi Wen, Elison B. Blancaflor

**Affiliations:** Noble Research Institute LLC, Ardmore, OK, United States

**Keywords:** truncated T-DNA, next generation sequence, forward genetics, software, HLB3

## Abstract

Transfer (T)-DNA insertions in mutants isolated from forward genetic screens are typically identified through thermal asymmetric interlaced polymerase chain reaction (TAIL-PCR), inverse PCR, or plasmid rescue. Despite the popularity and success of these methods, they have limited capabilities, particularly in situations in which the T-DNA is truncated. Here, we present a next generation sequencing (NGS)-based platform to facilitate the identification of complete and truncated T-DNA insertions. Our method enables the detection of the corresponding T-DNA insertion orientation and zygosity as well as insertion annotation. This method, called TDNAscan, was developed to be an open source software. We expect that TDNAscan will be a valuable addition to forward genetics toolkits because it provides a solution to the problem of causal gene identification, particularly genes disrupted by truncated T-DNA insertions. We present a case study in which TDNAscan was used to determine that the recessive *Arabidopsis thaliana hypersensitive to latrunculin B (hlb3)* mutant isolated in a forward genetic screen of T-DNA mutagenized plants encodes a class II FORMIN.

## Introduction

Forward genetics is an approach used to identify genes that control plant phenotypes of interest. Forward genetics involves screening a population of plants that has been mutagenized by chemicals, radiation, or biological agents. The latter method of mutagenesis involves *Agrobacterium tumefaciens*-mediated transfer-DNA (T-DNA) integration (Gelvin, 1998). T-DNA used in mutagenesis is modified from the original tumor-inducing (Ti) plasmid with 25 base pairs direct repeat border sequences at both ends (Zambryski et al., 1980; Hoekema et al., 1983). In the model plant species *Arabidopsis thaliana*, the modified T-DNA-based transformation system has been widely used not only to generate loss-of-function mutants via insertional mutagenesis but also for gene overexpression by activation tagging (Alonso et al., 2003; Rosso et al., 2003; Ichikawa et al., 2006). Because of the known sequences of the inserted T-DNA, recovering the insertion sites in the genome has become a routine operation. The process of insertion site recovery involves primarily polymerase chain reaction (PCR)-based methods, including thermal asymmetric interlaced PCR (TAIL-PCR) (Liu et al., 1995), inverse PCR (Ochman et al., 1988), or restriction site PCR (Ji and Braam, 2010). The relative ease by which T-DNA insertion sites are identified by PCR-based methods has also enabled the rapid development of reverse genetic resources in plants. In *Arabidopsis*, one can search for T-DNA insertions in a particular gene through stock centers in the USA, Europe, and Japan with the four most widely used mutant populations being SALK (Alonso et al., 2003), GABIKat (Rosso et al., 2003), SAIL (Sessions et al., 2002), and FLAGdb (Samson et al., 2002) lines. 

Despite the widespread implementation of T-DNA technology for forward and reverse genetics, there are cases in which causal gene identification can become problematic. Problems arise in part because of the complex mechanisms underlying T-DNA insertion, which to date remain poorly understood (Kleinboelting et al., 2015). One particular process that complicates identification of T-DNA insertion sites is the manner by which T-DNA is integrated into the plant genome. In many cases, T-DNA is not simply inserted into plant genomes as an intact unit. It has been reported that T-DNA can be truncated at the left and/or right ends before being inserted into the genome (Gheysen et al., 1990; Radhamony et al., 2005; Bartlett et al., 2014; Wu et al., 2014; Schouten et al., 2017). For example, Bartlett et al. (2014) reported that 7 out of 21 transgenic Barley lines were truncated with as much as 81 bp at the right border of the T-DNA sequence being lost. In another case, 26.5% of transgenic sorghum plants were discovered to have single-copy truncated T-DNA insertions (Wu et al., 2014). Finally, Schouten et al. (2017)identified a 50-bp fragment insertion that originated from the central part of a T-DNA construct in *Arabidopsis*. All of these examples make it likely that genetic mutations resulting from truncated T-DNA is a common occurrence in T-DNA mutagenized populations.

The current advancements in next generation sequencing (NGS) technologies have complemented PCR-based approaches for T-DNA site identification. DNA insertions have been successfully identified using NGS data in *Arabidopsis* (Lepage et al., 2013; Inagaki et al., 2015), rice (Daniela et al., 2013; Park et al., 2015), and maize (Williams-Carrier et al., 2010). All these methods are based on the following two processes. First, all short reads to reference and T-DNA/vector sequence are mapped. Second, the reads are divided into three subgroups: 1) reads that only map to the reference genome, 2) reads that only map to the vector sequence, and 3) reads that map to both the reference genome and vector sequence. The reads mapped to both reference and vector sequence are used to identify the T-DNA insertion. However, none of these NGS-based methods have been developed into a software package that can be used for routine T-DNA insertion identification. 

The identification of transposon insertion sites (ITIS) tool was recently developed to facilitate the ITIS (mainly *Tnt1* insertion) using NGS data in the model legume *Medicago truncatula* (Jiang et al., 2015). Even though both *Tnt1* and T-DNA are foreign DNA insertions, which can be integrated to plant genomes, their mechanisms of integration are totally different. *Tnt1* usually generates a 5-bp target-site duplication sequence on both sides of the insertion sites. On the other hand, T-DNA insertion usually results in an intact or truncated integration. ITIS has limited or no capabilities to detect truncated T-DNA insertions because the border regions of *Tnt1* are truncated and no informative (mainly soft-clipped) reads can be captured using its algorithm. The limitation in existing software to identify T-DNA insertion sites from NGS data motivated us to develop TDNAscan. The major advantage of TDNAscan lies in its ability not only to rapidly identify complete T-DNA insertions but also to accurately detect truncated T-DNA. The utility of TDNAscan was benchmarked from a series of data simulations and three real biological datasets. One real dataset presented in case study 1 was derived from published T-DNA mutants. The other two datasets illustrated in case study 2 and 3 are from actual T-DNA insertion mutants. The T-DNA mutant in case study 3 is called *hypersensitive to latrunculin B 3 (hlb3)* that we isolated in a forward genetic screen. Using TDNAscan, we determined that the disrupted gene in *hlb3* encodes the actin regulatory class II formin, AtFH20. To the best of our knowledge, TDNAscan is the first software to identify truncated T-DNA insertions in plant genomes and therefore provides a valuable addition to forward genetics toolkits.

## Data and Methods

### Data Simulation

T-DNA sequences from pSKI015 (6,743 bp) vector were randomly truncated using a Python program called TDNATruncate.py in our TDNAscan GitHub account. This program guarantees that around half of the T-DNA sequences are truncated and half of the T-DNA sequences are complete. The truncated T-DNA was created based on the following criteria: 1) randomly truncate the T-DNA on the left and/or right side and 2) the total length of truncated T-DNA should be larger than or equal to 50 base pairs.

**Figure 1:**
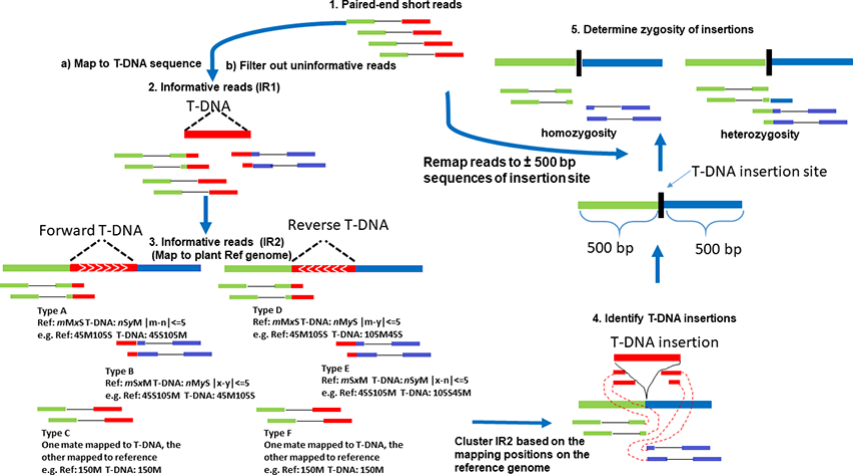
The flow chart of TDNAscan pipeline. Types A and B of soft-clipped reads are used to identify forward T-DNA insertions. Types D and E of soft-clipped reads are used to identify reverse T-DNA insertions, while types C and F of discordant reads are used to identify insertions but not the orientation of the insertions.

Using another Python program called TDNAInsert.py, 500 T-DNA sequences, including 237 complete T-DNA and 263 truncated T-DNA (see **Figure 1S**), were randomly inserted to *Arabidopsis thaliana* reference genome. 

Because paired-end reads are more informative than single-end reads in detecting structural variations, many bioinformatics tools were designed for paired-end reads analysis and benchmarked by paired-end simulated data. The most recent tools that use paired-end reads to identify deletion and insertions are FNBtools (Sun et al., 2018), ITIS (Jiang et al., 2015), VariationHunter (Hormozdiari et al., 2009; Hormozdiari et al., 2010) and BreakDancer (Chen et al., 2009). For our TDNAscan tool, simulated 150 bp paired-end NGS reads with 5x, 10x, 20x, and 40x coverage were generated using wgsim (Li et al., 2009) based on the T-DNA mutated reference genome from the Python program called TDNAInsert.py.

### Informative Reads Extraction

In our study, the discordant reads (DIR) are defined as one read of a pair successfully mapped to the plant reference genome and the other read of the same pair mapped to part of the inserted T-DNA. The soft-clipped reads (CLR) are reads where one partial read of a single read perfectly mapped to the plant reference genome and the other partial read of the same single read perfectly mapped to the inserted T-DNA. 

After mapping NGS reads to T-DNA or plant reference genome using BWA (Burrows–Wheeler Aligner) MEM (Li and Durbin, 2009), the output sequence alignment map (SAM) file is used to extract all informative reads. The Concise Idiosyncratic Gapped Alignment Report (CIGAR) strings from the SAM file for each read are important for detecting informative reads. Unlike ITIS, we mapped NGS reads to the T-DNA sequence first, which has smaller sequence size than the reference genome and then map all informative reads from the above step to plant reference genome. There are two types of informative reads in our pipeline. We defined CIGAR1 as the CIGAR strings from informative read (IR1) in the SAM file that was produced by NGS reads mapping to T-DNA. In turn, we defined CIGAR2 as the CIGAR strings from informative read in the SAM file that was produced by IR1 mapping to plant reference genome. All of the first set of informative reads (IR1) will be saved to align to the plant reference genome. The uninformative reads will be filtered out via parallel computing module. 

### Insertion Orientation and Truncated Position Detection

Information about the insertion orientation and truncated position is extremely important for biologists as they design PCR primers to experimentally confirm T-DNA insertions. TDNAscan can provide the truncated position at both side of the T-DNA sequence and the insertion orientation.

IR1 was used to align to plant reference genome via BWA MEM. Successfully mapped reads in IR1 were considered to be our second set of informative reads (IR2). IR2 and T-DNA insertion orientation were determined using six scenarios (**Figure 1**). Types A and B of CLR are used to identify forward T-DNA insertions. Types D and E of CLR are used to identify reverse T-DNA insertions, while types C and F of DIR are used to identify insertions but not the orientation of the insertions. The CIGAR2, for example, *m*M*x*S, is compared with CIGAR1, for example *n*S*y*M, where “M” and “S” in CIGAR1 and CIGAR2 represent sequence matches and sequence soft clipping, and “*n*” and “*y*” in CIGAR1 and “*m*” and “*x*” in CIGAR2 represent the number of sequence matches and soft-clipped nucleotides, respectively. All informative reads (IR1 and IR2) mapped to T-DNA and reference genome have to meet the following criteria: the total number of soft-clipped nucleotides for the same read mapping to T-DNA sequence should be the same or less than 5 bp difference from that of matched nucleotides mapped in reference genome (**Figure 1,** Step 3).

### Clustering of Informative Reads

All IR2 was represented by a sextuple (*chr, bp, read_type, tdna_border, tdna_pos*, and *orientation*), where *chr, bp, read_type*, and *orientation* represent chromosome, insertion position, read type (CLR and DIR), and the T-DNA insertion orientation (reverse or forward) of each informative read. The *tdna_border* and *tdna_pos* represent reads mapped to left or right border of T-DNA and their corresponding mapping position of T-DNA. All IR2 was clustered by the following steps: 1) sort all informative reads based on chromosome and insertion position (*bp*). For each chromosome, the sorted informative set is BP = (*bp_1_, …, bp_n_*). 2) Use the formula below to cluster the informative reads:

$$D_{i}=\;|bp_{i}-bp_{x}|\;\leq win$$

where 

$$\{\begin{array}{ll} bp_{x}=bp_{1}, & when\;i=2\\ bp_{x}=bp_{i-1,} & when\;i>2 \end{array}$$

We used window size 5 bp (*win* = 5) and 500 bp (library fragment length, *win* = 500) for CLR and DIR reads as default parameters, respectively, to cluster all informative reads for each chromosome. 3) If *D_i_* is satisfied, *bp_i_* will be added to cluster set C = (*bp_1_, …, bp_i_*) until *D_i_* fails. The position of this insertion will be determined using the following equation:

$$position=mode[C(bp_{1},\;\ldots ,bp_{i})]$$

Similarly, insertion orientation and T-DNA truncated position were determined by the same method. TDNAscan reports T-DNA insertions only if supporting reads ≥3 are observed.

### Zygosity Estimation

In forward genetics, the mutant lines are selfed or backcrossed with a wild-type plant in order to purify the mutant plants in a segregation population and confirm that the mutant phenotype is caused by one mutated gene. This is most likely disrupted by one homozygous T-DNA insertion. Knowing the zygosity will reduce the work load of researchers and allow them to only focus on the homozygous T-DNA insertions. We extracted 500-bp upstream and downstream sequences from each identified T-DNA insertion site and formatted the sequences to Insertion Candidate FASTA (ICF) sequences. All NGS data from step 1 in **Figure 1** were remapped to ICF sequences by BWA MEM. The reads with minimal mapping quality (MAPQ ≥ 30), which can span the T-DNA insertion sites at least 5 bp at both sides, will be considered as having the presence of a reference allele.

The T-DNA insertion frequency was determined by the following equation:

$$insertion\;frequency=\frac{N_{clr}}{N_{clr}+N_{span}}$$

*N*_clr_ is the number of CLR at the insertion region.

*N*_span_ is the number of reads spanning 5 bp upstream and downstream of the insertion site.

### Annotation of Identified T-DNA Insertion

In the long list of identified candidate T-DNA insertions, researchers always put more attention on the ones that are inserted to gene regions including 5’UTRs, 3’UTRs, introns, and especially exons. These types of insertions will disrupt gene function, which likely leads to mutant phenotypes. Annotation of identified T-DNA insertions will narrow the candidate insertions that are potentially causing mutant phenotype. This will accelerate the process of correctly discovering the causative genes. The Generic Feature Format version 3 (GFF3) file from species of interest in public genome databases [e.g., Phytozome (Goodstein et al., 2012)] have gene location information. TDNAscan uses GFF3 file to annotate all identified T-DNA insertions. If the genomic region of an identified T-DNA insertion overlaps with the region of annotated gene region in GFF3 file, this identified T-DNA insertion will be labeled with the gene ID in GFF3 file. TDNAscan software has a function to annotate all identified complete and truncated T-DNA insertions. Therefore, researchers can focus on T-DNA insertions, which fall on the gene regions and are most likely causal insertions of the mutant phenotypes.

### Case Study Samples and Sequencing

One T-DNA insertion pool, which consisted of five *Arabidopsis* SALK T-DNA insertion lines, was sequenced in case study 2. For each insertion line, the T-DNA identified by TDNAscan was confirmed by designing, an insertion-specific forward or reverse primer (depending on the T-DNA insertion orientation) to a region about 200–300 bp upstream (for the forward primer) or downstream (for the reverse primer) of the predicted insertion site. Meanwhile, a forward and a reverse primer were designed in the region between the left and the right border of the SALK T-DNA vector pROK2. Based on the predicted T-DNA insertion orientation, an insertion-specific forward (or reverse) primer in combination with a T-DNA reverse (or forward) primer was used to amplify a specific insertion fragment using the pooled DNA as the template. PCR was carried out using Ex-Taq as the DNA polymerase and a standard three-step program with the annealing temperature between 54 and 58°C and the extension time of 30–60 s. 

The mutant used in case study 3 to validate TDNAscan was isolated from an *Arabidopsis thaliana* activation-tagged T-DNA population (seed stock CS31100) obtained from the *Arabidopsis* Biological Research Center (ABRC, Columbus, Ohio). DNA from the mutant was extracted using Plant DNAzol Reagent (Invitrogen). Briefly, liquid nitrogen was used to grind 10-day-old seedlings into a fine powder, and the resulting material was mixed in an equal volume of Plant DNAzol reagent and chloroform. Following centrifugation, the aqueous phase was mixed with 100% ethanol to precipitate gDNA. The pellet was washed multiple times before being dissolved in water. The high-quality DNA sample was then sent for sequencing *via* Hi-Seq.

To genotype *hlb3-2* (SALK137002), the following primers were used: 137002LP-TTCCCTGAAGCCATTACACTG, 137002RP-GTAGCTCCATCTCCTCCTTGG, and LBb1.3-ATTTTGCCGATTTCGGAAC. To verify the insertion sites, the following primers were used: 37013F-GAAGAGGCTTACACCAGTTCTC, 37013R-GGAGGAGGAGGAGGATCATTA, 2465576F-GGAGGCAACAAAGTTTCACTG, 2465576R-TGAGCAGCGAAAGAGAAGAAC, 8582993F-GTTGTCATGTCTCCTCCAACTC, 8582993R-TCCTAACTGGCGCAACTATTC, tDNA1-GGCCGCTCTAGAACTAGTGG, tDNA2-CCACTAGTTCTAGAGCGGCC, tDNA3-CTAGATCTCGAGCTCGAGATC, and tDNA4-GATCTCGATCTCGAGATCTAG.

## Results and Discussion

**Figure 1** shows the flow chart of TDNAscan pipeline. Five steps were used to develop TDNAscan software (see Methods). 1) Paired-end short reads were first mapped to T-DNA sequences via BWA MEM. 2) The first informative reads (IR1) were extracted, and noninformative reads were filtered out. To accelerate this process, the Python multiprocessing library was used to parallel process this step (the default core is 8 cores). 3) IR1 were then mapped to the plant reference genome via BWA MEM again. Only the informative reads (IR2) that can both mapped to T-DNA and reference genome were used for clustering analysis. 4) T-DNA insertions were identified based on clustering analysis of IR2. 5) Lastly, all NGS reads were remapped to the ±500 bp sequences of identified insertion sites. Zygosity of T-DNA insertions was estimated. 

### Benchmarking TDNAscan on Simulated Data

TDNAscan has been benchmarked on simulated data. Simulated 150-bp paired-end NGS reads with 5x, 10x, 20x, and 40x coverage were used to test the accuracy of TDNAscan. Identified T-DNA insertions using TDNAscan are only considered as true insertions when their insertion positions are ±100 bp from the position of true T-DNA insertions. We measured the recall and precision values together with the accuracy score, F-score, described below:

$$\text{Precision}=\frac{TP}{TP+FP}\;\mathrm{TP}:\mathrm{truepositive};\;\mathrm{FP}\text{​}:\text{​ }\mathrm{false}\;\mathrm{positive}$$

$$\text{Recall}=\frac{TP}{TP+FN}\;\mathrm{TP}:\mathrm{truepositive};\;\mathrm{FN}:\;\mathrm{false}\;\mathrm{negative}$$

$$\text{F-score}=2*\frac{Precision*Recall}{Precision+Recall}$$

**Figure 2:**
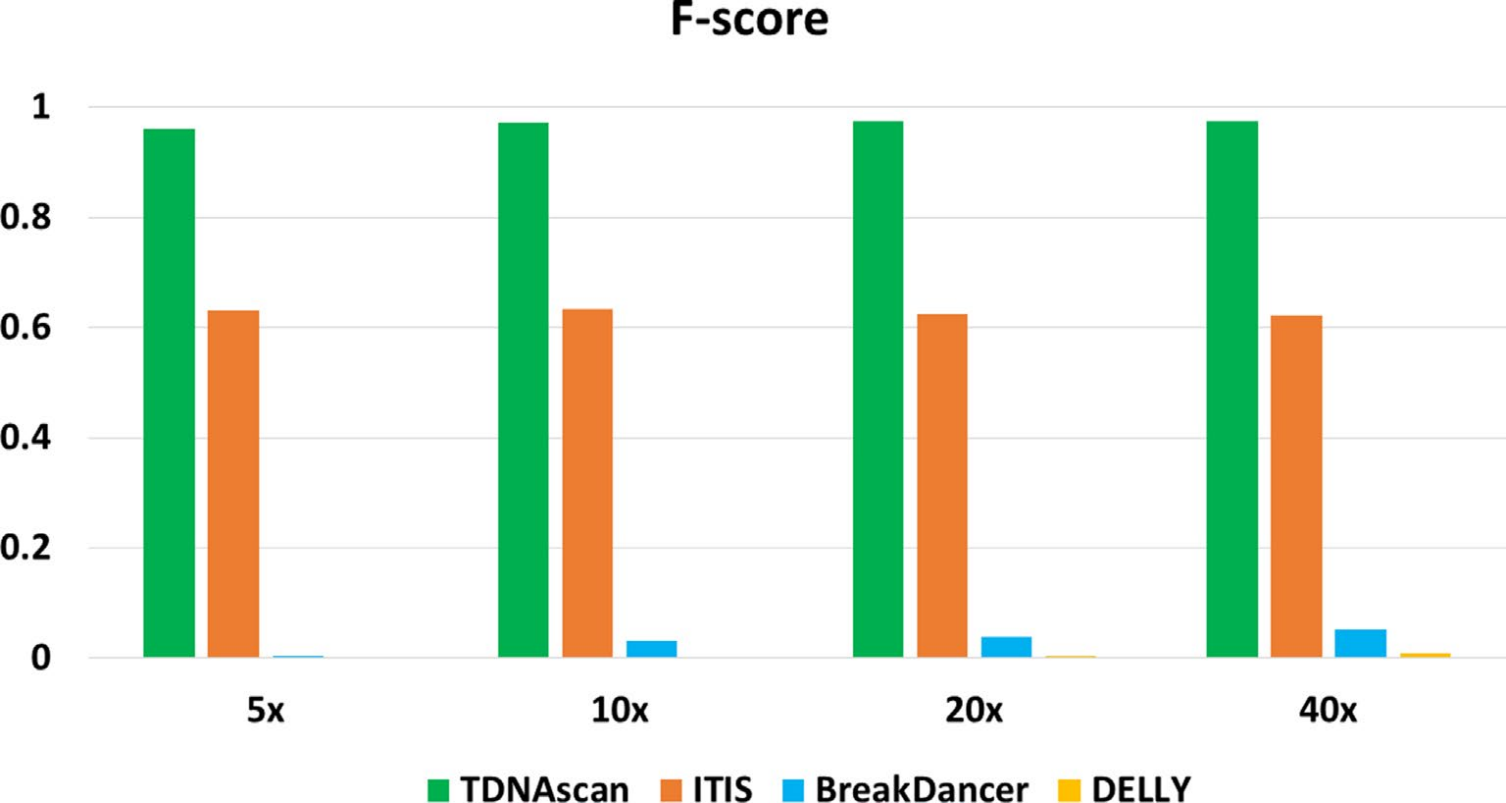
The accuracy of TDNAscan and similar tools with different coverage simulated data. The green, orange, blue, and yellow bars represent the F-scores of TDNAscan, ITIS, BreakDancer, and DELLY, respectively.

F-scores for simulated data at 5x, 10x, 20x, and 40x coverage were 0.96, 0.972, 0.975, and 0.974, respectively (**Figure 2** and **Table 1**). To help users choose the length of paired-end reads and coverage of NGS data in their projects, we also benchmarked TDNAscan for 100- and 300-bp paired-end data at 2x, 5x, 10x, 20x, and 40x coverage, respectively. Based on our simulated results (**Supplementary file 1**), TDNAscan achieved the highest accuracy when the read length of simulated NGS data is 300 bp. TDNAscan can also handle as low as 2x coverage data with at least 66.4% accuracy in our simulated data (**Supplementary file 1**).

**Table 1:** Comparison of similar tools.

Tools	5x F-score		10x F-score		20x F-score		40x F-score		Insertion orientation	Zygosity	Insertion annotation
	M^1^	N^2^	M	N	M	N	M	N			
TDNAscan	**0.960**		**0.972**		**0.975**		**0.974**		✓	✓	✓
	465	241	477	250	479	253	479	254			
BreakDancer	0.004		0.031		0.039		0.053		⨯	⨯^3^	⨯
	1	1	8	8	10	7	14	11			
ITIS	0.631		0.634		0.624		0.623		✓	✓	⨯
	236	11	240	12	236	8	236	9			
DELLY	0		0		0.004		0.008		⨯	⨯	⨯
	0	0	0	0	1	1	2	2			

^1^The total number of true complete and truncated T-DNA insertions identified.

^2^The total number of true truncated T-DNA insertions identified.

^3^The BreakDancer author claimed that the allele frequency is not accurate and should not be trusted.

### Comparison With Similar Tools

Even though there are no similar bioinformatics tools that can identify complete and truncated T-DNA insertions, we did find tools that can identify complete T-DNA insertions albeit with a limited accuracy. We compared our TDNAscan tools with ITIS (Jiang et al., 2015), DELLY (Rausch et al., 2012), and BreakDancer (Chen et al., 2009) in F-score, the capability of identifying insertion orientation and zygosity. Both BreakDancer and DELLY were not able to use the known T-DNA sequence information. Therefore, they can rarely detect T-DNA insertions. ITIS has limited ability to detect complete T-DNA and is not capable of detecting truncated T-DNA. TDNAscan outperforms other tools in F-score; specifically, it outperforms in the area of detecting truncated T-DNA insertions (**Figure 2** and **Table 1**). In addition, TDNAscan is the only tool that provides annotation function of identified T-DNA insertions (**Table 1**).

To demonstrate the utility of TDNAscan, we present three case studies using published data and actual NGS data from *Arabidopsis* mutants.

## Case Study 1

**Figure 3:**
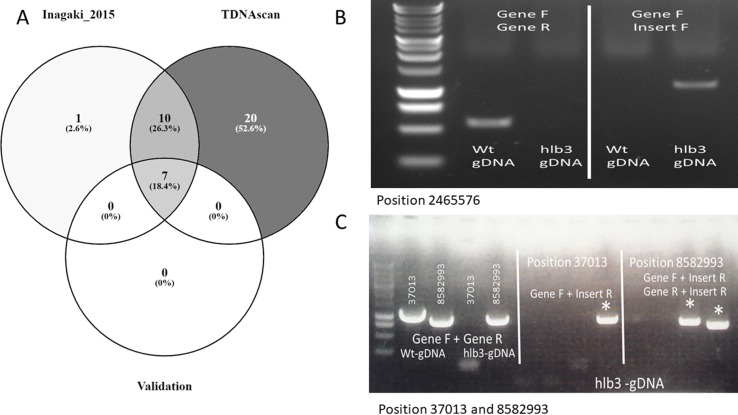
The graph of case studies. **(A)**. Venn diagram of identified T-DNA insertions in published data. **(B)**. PCR validation of predicted T-DNA insertion at location 2465576, the causative insertion of *hlb3*. **(C)**. PCR confirmation of the remaining two insertion sites to show further validation of the TDNAscan prediction.

To validate the performance of our TDNAscan, we used 20 transgenic lines of *Arabidopsis thaliana* with four different T-DNA vectors for each line from Inagaki et al. to test TDNAscan (Inagaki et al., 2015). All NGS data were downloaded from NCBI sequence read archive (SRA) database (BioProject ID: PRJNA287142 and SRA ID: SRP059868) using SRA toolkitv2.9.2 (Leinonen et al., 2011). Low-quality NGS reads were trimmed and discarded using Trimmomatic v0.35 (LEADING:3 TRAILING:3 SLIDINGWINDOW:4:20 MINLEN:36) (Bolger et al., 2014). In total, we successfully identified 37 T-DNA insertions using TDNAscan. Inagaki et al. used DIR and self-written Python script to identify 18 T-DNA insertions. Compared with these 18 T-DNA insertions, we successfully identified 17 T-DNA and additional truncated T-DNA insertions (**Figure 3A** and **Supplementary File 2**). With the seven experimentally validated T-DNA insertions by Inagaki et al., we identified all of them via TDNAscan. Based on this validation data, the T-DNA insertion prediction by TDNAscan is all accurate.

## Case Study 2

For case study 2, we used actual *Arabidopsis* SALK T-DNA insertion lines to validate the TDNAscan software. Whole genome Hi-Seqillumina paired-end reads, which were deposited into NCBI SRA database (BioProject ID: PRJNA 539954), were trimmed via Trimmomatic v0.35 (LEADING:5 TRAILING:5 SLIDINGWINDOW:4:30 MINLEN:40) (Bolger et al., 2014). Approximately 124.5 million high-quality reads were used to identify T-DNA insertions via TDNAscan software. 12 T-DNA insertions were identified using TDNAscan. Out of these 12 insertions, 11 insertions (91.7%) were confirmed by PCR in the pooled DNA samples (**Supplementary File 3**). We were unable to validate one insertion (Insert-4) by PCR because it had no soft-clipped supporting reads (no exact insertion position) and low frequency (close to 0) in the pool. To further study the reads and mapping status of the nonvalidated insertion, we used the Integrative Genomics Viewer (IGV) (Thorvaldsdottir et al., 2013) to visualize all informative read alignments. Based on our IGV analysis, we speculated that this T-DNA insertion (Insert-4) caused a copy number variation near the insertion site, where both the truncated T-DNA sequence and genomic sequence were copied several times. Copy number variation can interfere with PCR-based validation techniques because primers cannot specifically bind to the repeated genomic regions. Overall, our TDNAscan software achieved 91.7% accuracy to identify T-DNA insertions in actual *Arabidopsis* SALK T-DNA insertion lines.

## Case Study 3

**Figure 4:**
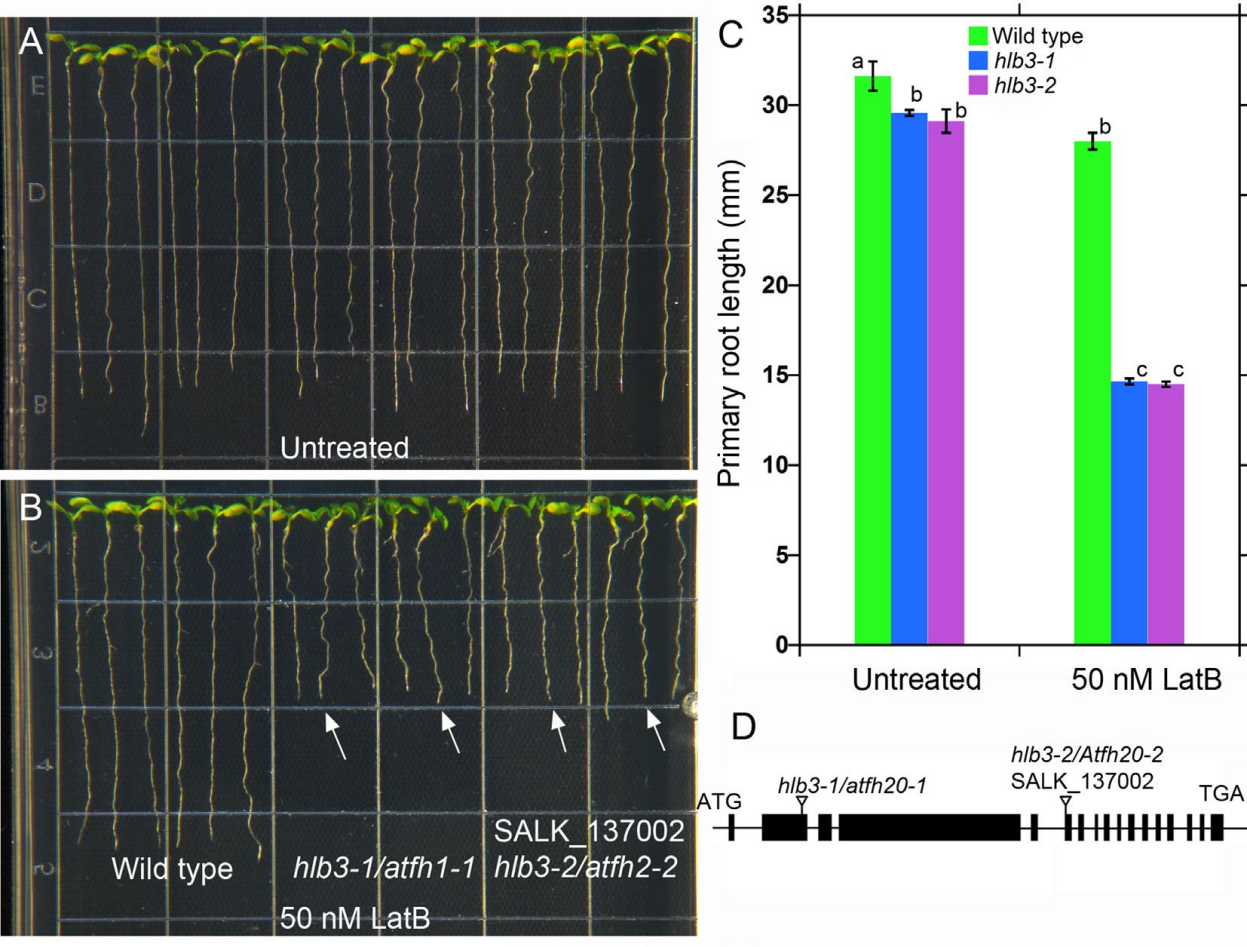
The *Arabidopsis HLB3* gene encodes the class II formin AtFH20. **(A–B)** The *hlb3-1/atfh20-1* and *hlb3-2/atfh20-2* mutants exhibit heightened sensitivity to the root growth inhibitory effects of latrunculin B when compared to wild type (arrows in **B**). Four-day-old seedlings of all genotypes were transferred to plates containing 50 nM latrunculin B and imaged after 3 days. **(C)** Quantification of primary root displacement after transfer to latrunculin B. Statistical significance was determined by one-way ANOVA. Means (*n* > 40) ± SE. Different letters indicate significant differences among means (*P* < 0.05, Tukey’s test). **(D)** Schematic diagram of the *HLB3/AtFH20* (*AT5G07740*) gene. Black boxes indicate exons, and lines indicate introns. Inverted triangles show the position of T-DNA for *hlb3-1/atfh20-1* and *hlb3-2/atfh20-2*.

We next asked whether TDNAscan can be used to identify the disrupted gene in the *Arabidopsis hypersensitive to latrunculin B* (*hlb3*) mutant. The recessive *hlb3* mutant exhibits heightened sensitivity to the actin-disrupting drug, latrunculin B, and was isolated from the same T-DNA mutagenized population as in our previously published *hlb1*mutant (**Figure 4A–C**) (Sparks et al., 2016).

Whole genome Hi-Seqillumina data from*hlb3*, which were deposited into NCBI SRA database (BioProject ID: PRJNA 539954), were trimmed via Trimmomatic v0.35 (LEADING:3 TRAILING:3 SLIDINGWINDOW:4:20 MINLEN:36) (Bolger et al., 2014). We used all 102.2 million high-quality paired reads and default TDNAscan parameters to successfully identify three T-DNA insertions in *hlb3* (**Table 2**). TDNAscan revealed that all identified T-DNA insertions in *hlb3* were truncated. These three identified truncated T-DNA insertions, with correct orientation and zygosity, were validated via PCR. For T-DNA truncation validation, we used primers specific to the inserted T-DNA sequence and to that of the predicted insertion site in the *Arabidopsis* genome. We were able to amplify the three chimeric products and verify the junctions between the T-DNA and genomic DNA via sequencing (**Figure 3B, C**).

**Table 2:** T-DNA insertions identified by TDNAscan in a real case study 2.

Chromosome	Position	Informative Reads	T-DNA truncation	Strand	Freq	Annotation
Chr5	37013	CLR:117,DIR:44	tdna_st:16,tdna_end:-	−	1	AT5G01100
Chr5	2465576	CLR:4,DIR:59	tdna_st:-,tdna_end:6442	+	1	AT5G07740
Chr5	8582993	CLR:147,DIR:59	tdna_st:23,tdna_end:-	+	0.7	–

CLR represents the total number of soft-clipped reads in the insertion region; DIR represents the total number of discordant reads in the insertion region; tdna_st represents the start position of T-DNA sequence after insertion. tdna_end represents the end position of T-DNA sequence after insertion.

Two of the three truncated T-DNA insertions were located in the exon 2 of genes *AT5G07740* (**Figure 4D**) and intron 7 of *AT5G01100*. *AT5G07740* is annotated to encode a FORMIN Homology 20 (AtFH20) protein, while *AT5G01100* encodes a O-fucosyltransferase family protein. The former belongs to a large family of proteins that play an important role in the organization of the actin cytoskeleton. Based on the phenotype of *hlb3* and the location of the truncated T-DNA in the exon region of *AT5G07740*, we hypothesized that disruption in the *ATFH20* gene is the cause of the *hlb3* phenotype. To test this hypothesis, we obtained a SALK (SALK_137002) line (see Methods) that had a T-DNA insertion on the *AT5G07740* gene from the ABRC. We verified that SALK_137002 had a T-DNA insertion in the sixth exon of *AT5G07740* (**Figure 4D**). Like *hlb3*, SALK_137002 exhibited enhanced growth inhibition to latrunculin B compared to wild type, indicating that the *HLB3* is *AtFH20* (**Figure 4A–C**). Based on our findings, the *hlb3* was renamed *hlb3-1/Atfh20-1*and SALK_137002 as *hlb3-2/Atfh20-2* (**Figure 4D**). Taken together with simulation data, identification of the truncated T-DNA in the *HLB3* gene demonstrates the utility of TDNAscan software for causal gene identification in mutants isolated from T-DNA mutagenized populations.

## Conclusion

We successfully developed a bioinformatics software named TDNAscan to identify complete and truncated T-DNA insertion in *Arabidopsis thaliana* mutant population. In addition to T-DNA insertion identification, this tool provides essential information such as orientation, zygosity, and annotation of the identified T-DNA insertion. The strength of TDNAscan lies in its ability to detect truncated T-DNA insertions, a feature not available with existing software. Although validation of the TDNAscan software as reported here is limited to *Arabidopsis*, we plan to implement it in other T-DNA insertional mutants from other plant species once NGS data becomes available.

## Data Availability

The datasets GENERATED for this study can be found in NCBI https://www.ncbi.nlm.nih.gov/Traces/study/?acc=PRJNA539954.

## Author Contributions

LS and EB conceived the original research plans. LS developed the bioinformatics tools. YG and ZR performed simulation data analysis. XC and JW performed case study 2. JAS performed case study 3. LS and EB supervised and wrote the original draft. EB, JW, and LS reviewed and revised the writing.

## Funding

This work was supported by ESI Innovation Project of Noble Research Institute and in part by National Aeronautics and Space Administration (NASA grant numbers 80NSSC18K1462 and 80NSSC19KO129) to EB.

## Conflict of Interest Statement

The authors declare that the research was conducted in the absence of any commercial or financial relationships that could be construed as a potential conflict of interest.

## Supplementary Material

Figure S1 The distribution of truncated T-DNA length on simulated data.
