# Supplementary figures and images for: TDNAscan: A Software to Identify Complete and Truncated T-DNA Insertions
Source: Front Genet. 2019 Jul 25;10:685. doi: 10.3389/fgene.2019.00685 (PMC6690219; doi:10.3389/fgene.2019.00685)

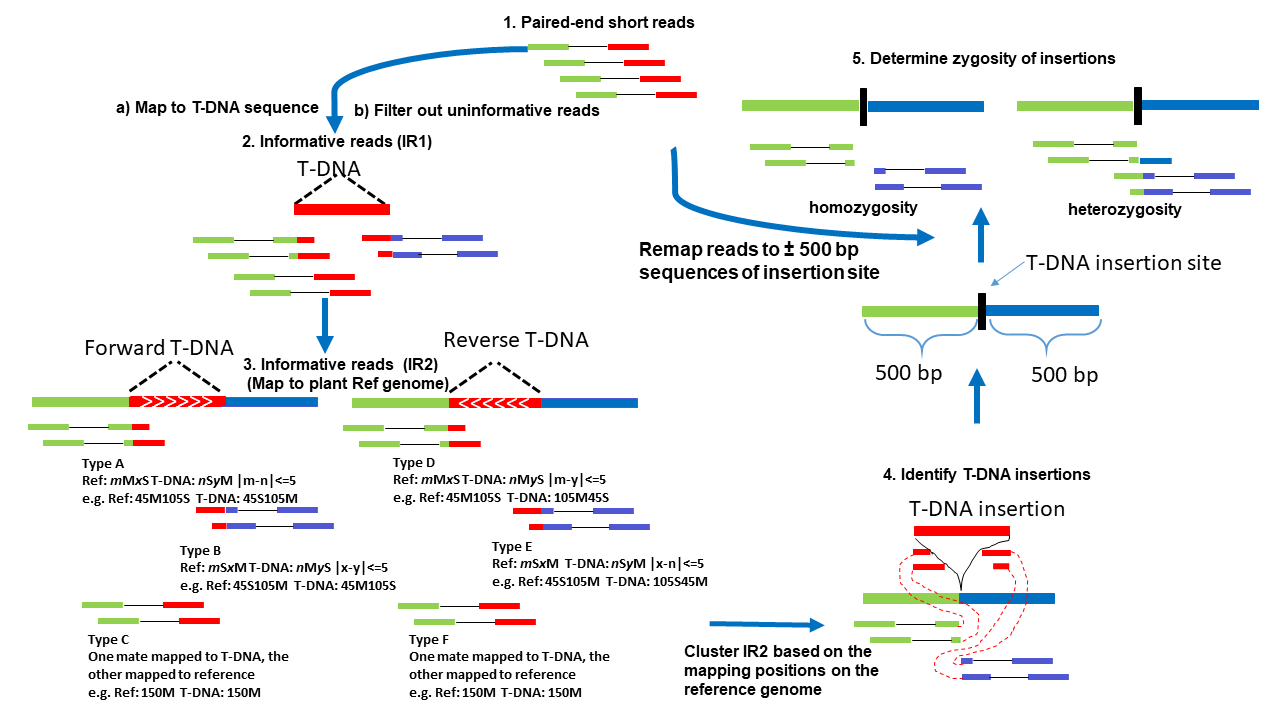

Supplement: Figure S1 — The distribution of truncated T-DNA length on simulated data. [file Image_1.tif]

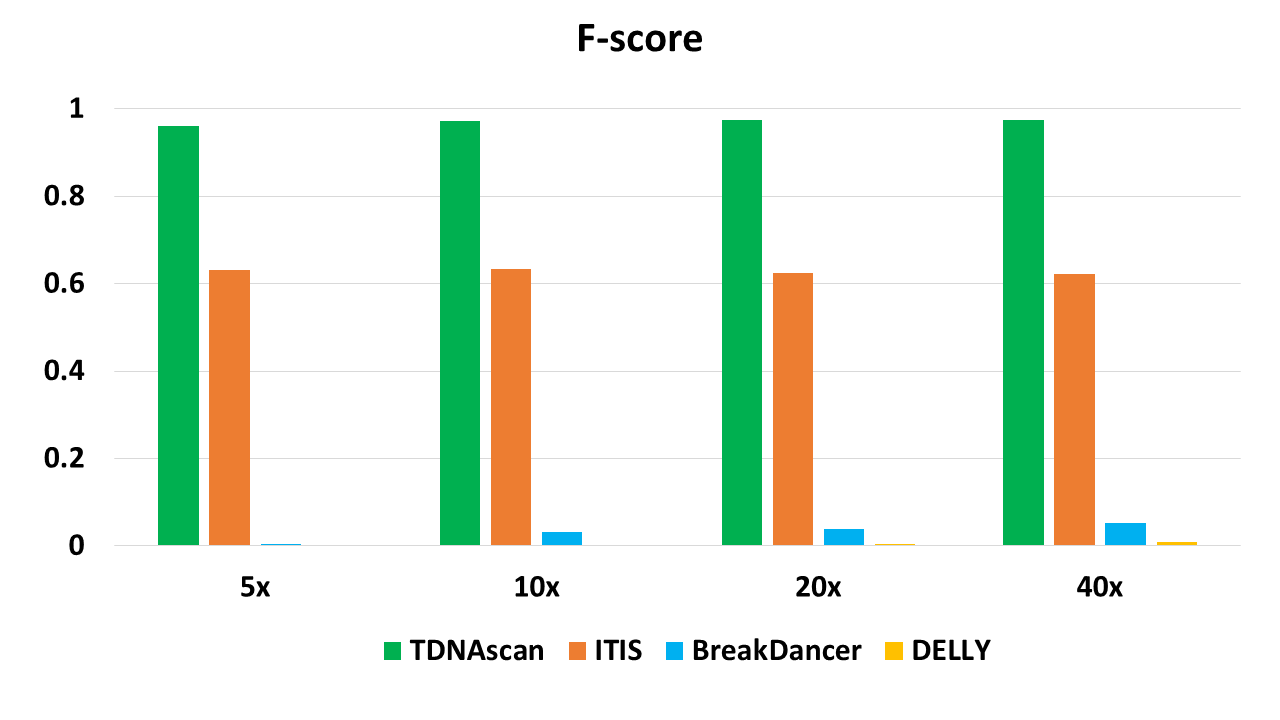

Supplement: Supplementary file 2 [file Image_2.tif]

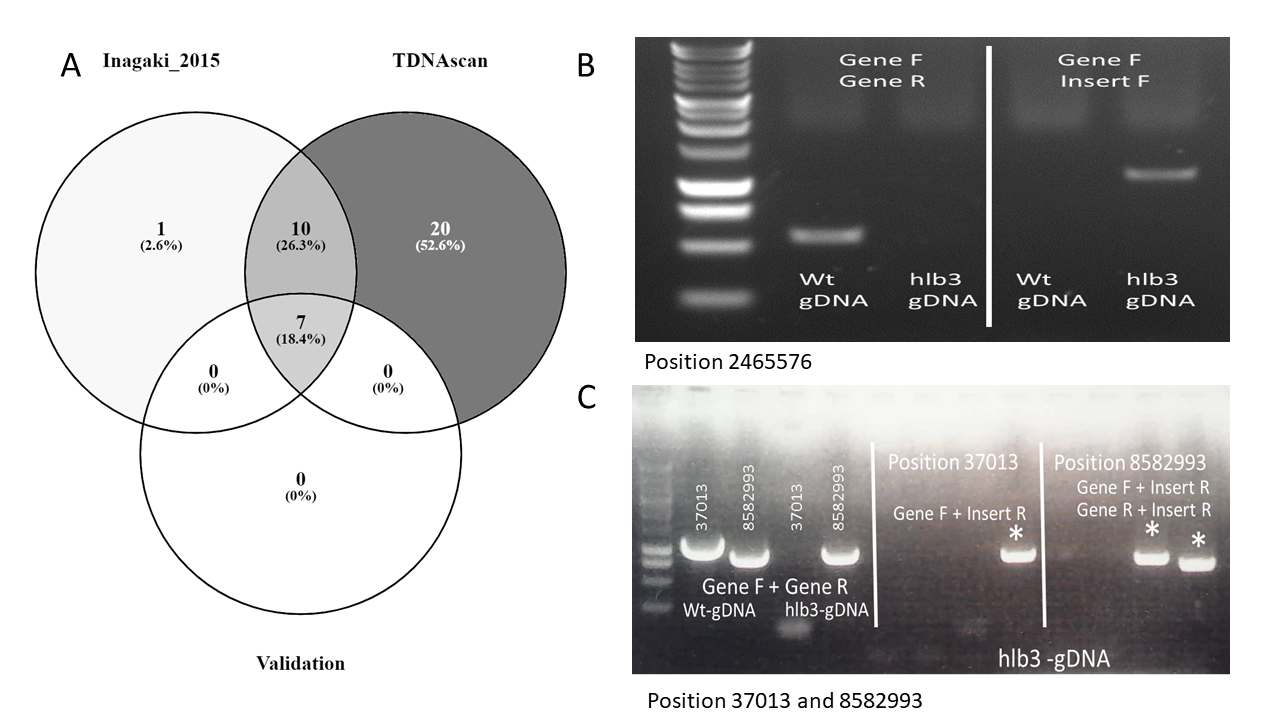

Supplement: Supplementary file 3 [file Image_3.tif]

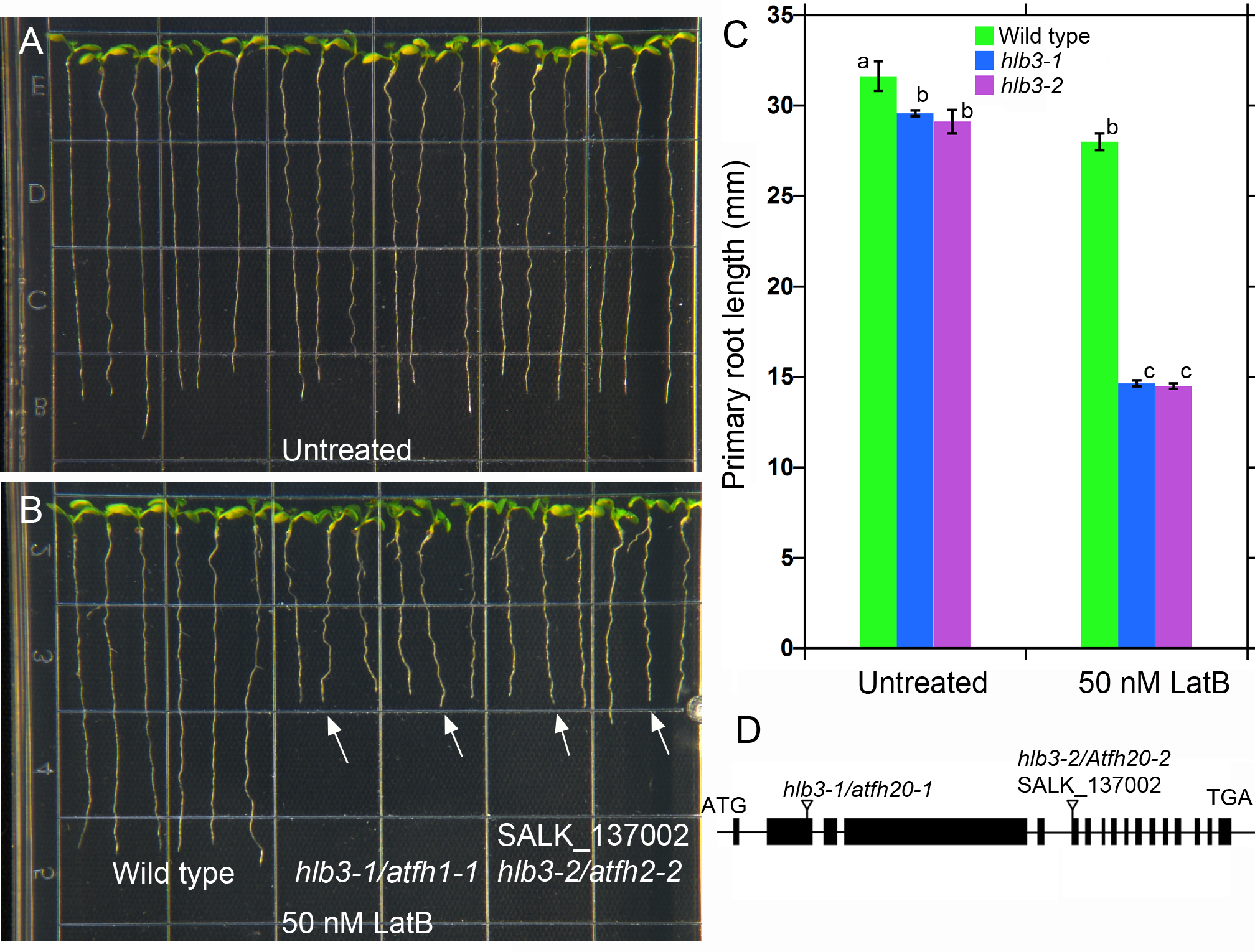

Supplement: Supplementary file 4 [file Image_4.tif]
